# Evaluation of bond strength of orthodontic brackets without enamel etching

**DOI:** 10.4317/jced.52253

**Published:** 2015-10-01

**Authors:** Alireza Boruziniat, Yegane Khazaei, Shiva Motaghi, Mohmmadjavad Moghaddas

**Affiliations:** 1 Assistant Professor of Restorative Dentistry, Dental Research Center, School of Dentistry, Mashhad University of Medical Sciences, Mashhad, Iran; 2 Under-graduate student, Student Research Center, School of Dentistry, Mashhad University of Medical Sciences, Mashhad, Iran; 3 Associated Professor of Restorative Dentistry, Dental Research Center, School of Dentistry, Mashhad University of Medical Sciences, Mashhad, Iran

## Abstract

**Background:**

To compare the shear bond strength of brackets with and without enamel etching.

**Material and Methods:**

In this study, 60 sound premolars were randomly divided into four different groups: 1- TXE group: Enamel etching+Transbond XT adhesive+ Transbond XT composite. 2- TXS group: Transbond plus self-etch adhesive+ Transbond XT composite. 3- PQ1E group: Enamel etching+ PQ1 adhesive+ Transbond XT composite. 4- PQ1 group: PQ1 adhesive+ Transbond XT composite. The shear bond strengths of brackets were evaluated using universal testing machine at cross head speed of 0.5 mm/min. The Adhesive Remnant Index (ARI) was also measured. One-way ANOVA, Tukey’s post hoc, Kruskal-wallis and Mann-Witney U test were used for data analysis.

**Results:**

There was a significant difference between etched and unetched groups respect to SBS and ARI (*p*<0.05), however; no significant difference was observed between unetched group and self-etch adhesive group (*p*>> 0.05). The shear bond strength of PQ1 group was the least but in acceptable range and its ARI was less than other groups.

**Conclusions:**

PQ1 adhesive can be used for bracket bonding without enamel etching with adequate bond strength and minimal ARI.

** Key words:**Bracket, shear bond strength, filled-adhesive, self-etch adhesive.

## Introduction

Acid etching technique was first introduced by Bounocor for an improved bonding to the tooth structure in 1955. Since then,there has been magnificent progress in direct bonding of orthodontic brackets. In late 1970, the cementation of orthodontic band, which have brackets on itself,was replaced with direct bonding of brackets to the enamel (1). The traditional method for the bonding of brackets is the application of total-etch adhesive systems include cleaning the enamel surface, application of acid phosphoric, rinsing and drying, applying adhesive system and composite resin. Although this method is remarkably effective, it has few disadvantages as follow: the process is time-consuming and some areas of etched enamel surface are not fully covered with adhesive which may lead to the formation of white spots. White spots may cause unpleasant appearance after orthodontic treatment (2). The application of total-etch technique provides high bond strength, therefore it can cause enamel fracture during the debonding, more amounts of residual resin on enamel surface and more chair time for removing the remnants. Furthermore, more enamel may be lost during the elimination of residual resin (3-5).

The value of bond strength plays an important role in the bonding of brackets. Reynolds *et al.* (6) found that minimum amount of bond strength for resistance to debonding is between 5.9 to 7.8 MPA.

Despite the fact that efficient bond strength for orthodontic brackets is necessary for orthodontic treatments, but it should be considered that the bond is temporary and the brackets will be removed at the end of treatment process (7). Therefore, one of the primary goals is to preserve the enamel surface sound and unchanged after the orthodontic treatment (8).

Different methods have been purposed to minimize the drawbacks of traditional total-etch technique. Some studies showed that the bond strength of orthodontic brackets obtained from lower concentrations of phosphoric acid (2%, 5%, 10%) has no significant difference with 37% acid phosphoric (9,10).

Osorio *et al.* (11) reported that the shear bond strength obtained after 60s etching was higher than those etched for 30s, however; there is more amount of residual resin on enamel surface after removing the brackets.

For the aim of reducing the enamel loss during etching, Bishara *et al.* (12) used 10% polyacrylic acid and glass ionomer adhesive for bonding the brackets, concluding that the application of glass ionomer adhesive may decrease decalcification around the brackets. Nevertheless, the shear bond strength was relatively lower than those etched with acid phosphoric and bonded with composite.

Several studies suggested that self-etch primers (SEPs) are suitable alternatives to total-etch technique and demonstrated that there is no significant difference between bond strength obtained from these methods (13,14). Some advantages of SEPs include simplicity of application, less chair-time, establish minimum etched pattern which can cause adequate bond strength, less residual resin on enamel surface after bracket debonding, faster and easier resin removal with less damage to enamel surface (15-17).

Different researches showed that adding filler to adhesives can improve the mechanical properties such as flexural strength and modulus (18). In addition, filled-adhesives have less polymerization contraction in comparison with unfilled-adhesives and can act as stress breaker against polymerization stress of composite which placed on it. Faltermeier *et al.* (19) found that filled-adhesives increase the bond strength of stainless steel brackets in comparison with those without filler. As the filler content of adhesives increased, more bond strength was obtained, although no significant difference was found in ARI. Also, Ostertag *et al.* (20) showed that the higher amounts of filler in adhesives increase the bond strength of ceramic brackets.

The initial authors’ assumption was that application of filled-adhesive without etching the enamel may provide adequate bond strength for bracket in clinical situations. Therefore the purpose of this study was to evaluate the bond strength of brackets to enamel by using filled-adhesive with or without enamel etching and comparing it with the traditional total-etch adhesive technique and self-etch primer application. The null hypothesis of this study was that there were no detectable differences in ARI and bond strengths of brackets obtained from filled-adhesive, self-etch primer and traditional total-etch adhesive.

## Material and Methods

Sixty premolar teeth without enamel hypoplasia, fractures, or caries, which had been extracted for orthodontic purposes, were collected for this study. The teeth were cleaned using some fluoride-free pumice slurry,were then mounted onselfcure acrylic resin blocks up to 1mm below the CEJ (Cement Enamel Junction) and kept in distilled water. The samples were randomly divided into four groups based on the bracket bonding methods applied:

Group 1 (TXE): At first, the middle third of buccal surfaces were etched for 30s using 37% phosphoric acid (Kimia, Iran), then rinsed and dried. Transbond XT primer (3M Unitek, California; USA) was rubbed for 10 seconds on the etched surface. Next, Transbond XT composite resin was applied on the bracket base (Ortho-organizer, California; USA) and the bracket was placed on the tooth. The excess of the composite was removed from around the base of the bracket before each of the four aspects was cured for 10s using light cure unit (Fast cure, Dental produd, USA).

Group 2 (TXS): Transbond Plus self -etching primer was rubbed on the mid-buccal surface for 3-5 seconds. Then Transbond XT composite resin was applied on the base of the bracket with the same procedure as the former group.

Group 3 (PQ1E): The enamel of buccal surface was etched using 37% phosphoric acid (Kimia, Iran), and procedure was followed by rinsing and drying. PQ1 adhesive (Ultradent, St. Louis; USA) was applied on the surface for 10s, gently air-dried and thinned for 10s and finally cured for 10s. The procedure followed the same pattern as in group 1.

Group 4 (PQ1): The procedure was similar to group 3 except the application of acid etch on enamel.

The shear bond test was performed using universal testing machine (Zwick/Z250, Type KAP-Z, ZwickRoellGroup; Ulm, Germany) at a crosshead speed of 0.5 mm/min. Enamel surfaces of specimens were examined after debonding procedure with a stereo-microscope (LEO, 1450 UP, Zeiss; Oberkochen, Germany) at ×10 magnification to determine the amount of residual adhesive remaining on each tooth (ARI).

ARI scores were given as follows:

0: No adhesive remained on tooth.

1: Less than 50% of adhesive remained on tooth.

2: More than 50% of adhesive remained on tooth.

3: The entire adhesive remained on tooth.

Data were analyzed by One-way ANOVA, Tukey’s post huc, Kruskal-Wallis and Mann-Whitney tests using SPSS16 software.

Results

Kolmogrov-Smirnov test showed normal distribution of data at pre-set significance level of 0.05. The mean shear bond strengths of experimental groups are presented in  Table 1. One-way ANOVA demonstrated a significant difference among the groups (*P*<0.05).

**Table 1:**
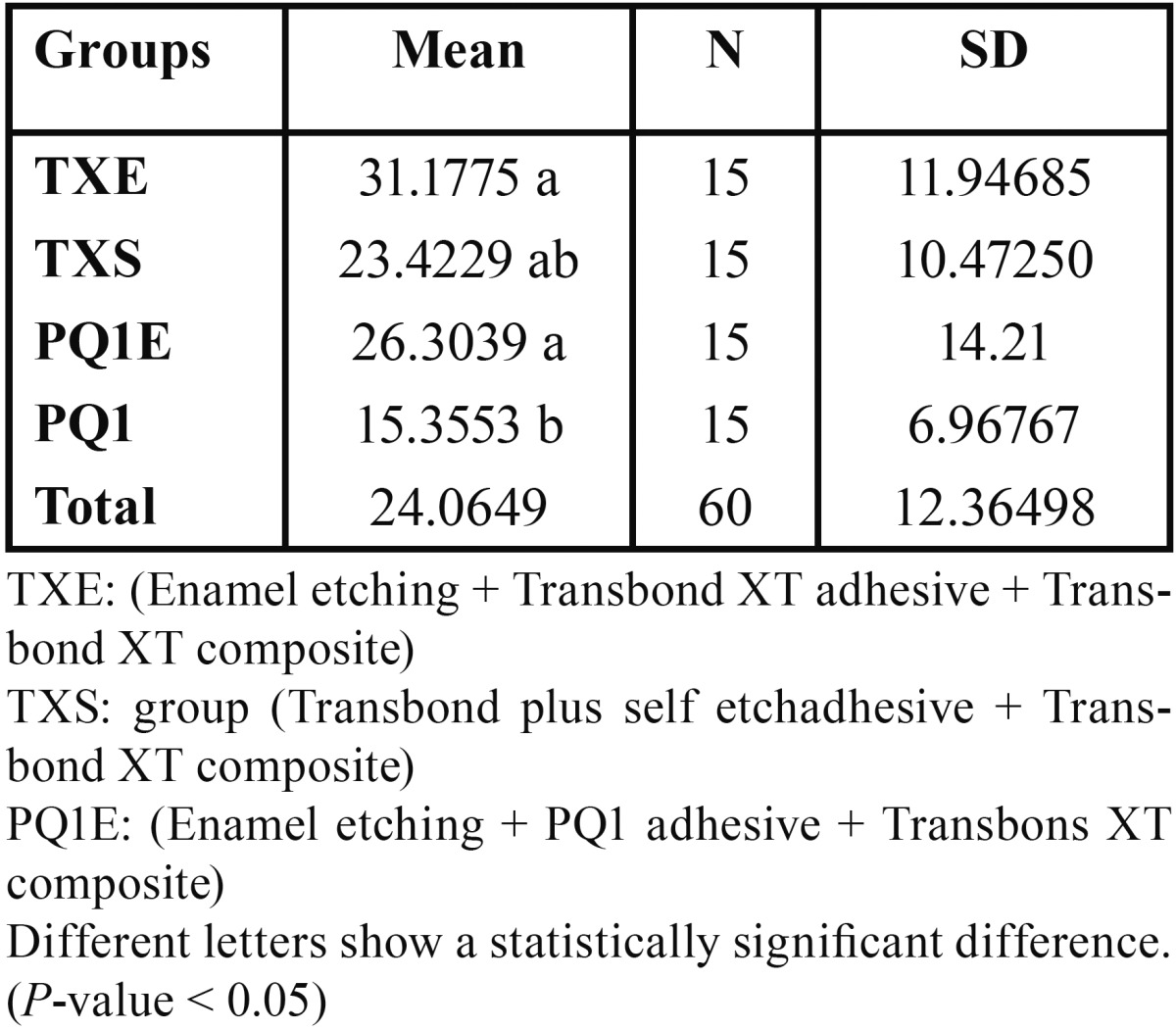
The mean value of shear bond strength and standard deviation of experimental groups.

Tukey’s post-hoc test revealed that while there were no significant differences among TXS, PQ1E and TXE groups, statistically significant differences could be observed when comparing PQ1 group with PQ1E and TXE groups (*P*< 0.05).

The mean ARI values of the experimental groups are presented in  Table 2. Krukal-wallis test showed a statistically significant difference among the experimental groups at pre-set significance level of 0.05. Mann-Whitney test indicated that the mean value of ARI of the PQ1 group was significantly lower than those of the other groups (*P*-value < 0.05) and that there was no statistically significant difference in the mean value of ARI among other experimental groups (*P*-value > 0.05).

**Table 2:**
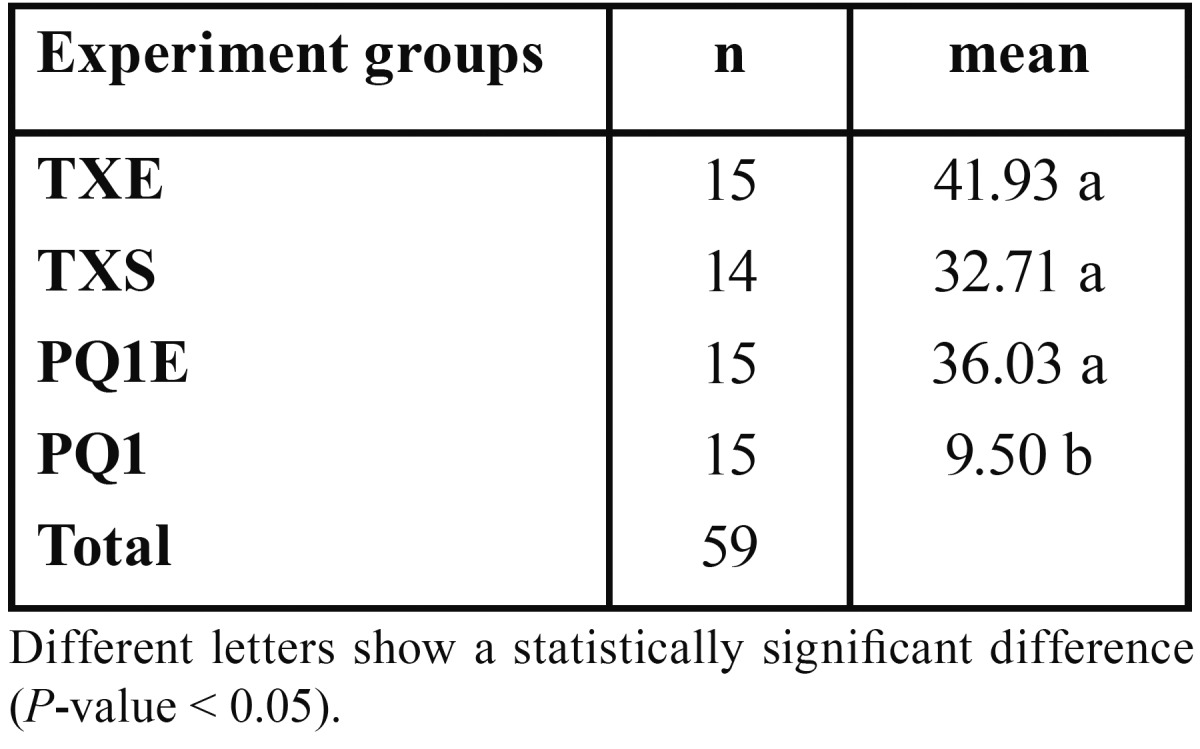
The mean value of ARI of experimental groups.

## Discussion

The results of the current study rejected the null hypothesis.

When PQ1 was applied without using the acid-etch, the mean values of bond strength and ARI decreased significantly in comparison with procedures involving acid-etch or routine methods of bracket bonding. However, it is necessary to mention that the bond strength of this filled-adhesive, with no etching step, was higher than the minimum bond strength which is required for ort-hodontic bracket bonding. The bond strength should be sufficient to resist masticatory forces and stresses caused by orthodontic wires. Conditioning of the teeth, adhesive systems, size, shape, and quality of the attachment, type of the teeth, bonding procedu-res, as well as the experience level of the operator are the important factors which may affect the bond strength (21).

Even though application of acid-etch, as a surface treatment agent, on enamel is an accepted method, it also has some disadvantages, which include: being a multi-step and time-consuming procedure, causing enamel decalcification (which can in turn develop enamel caries beneath the orthodontic attachments), and increasing the chances of enamel fracture during the debonding process. Moreover, the extent of damage to the enamel may increase while removing the remnants of resin from the surface of tooth, since the application of acid-etch technique is likely to leave more resin remnants after debonding of brackets. Thus using a simple technique which can provide acceptable bond strength would be desirable.

Several attempts have been made with the aim of establishing a technique which can induce maximum bond strength while minimizing enamel loss. For instance, Carstensen *et al.* (22) illustrated that 2% concentration phosphoric acid could generate more bond strength for metal brackets and lesser resin remnants on the surface of enamel in comparison with 38% phosphoric acid. However, according to their study, the application of 40% phosphoric acid produces even rougher surface but lesser resin remnants in comparison with 2% concentration of this acid. When the lower concentration of phosphoric acid was applied, the depth of decalcification, the enamel loss and the incidence caries were observed to decrease (22).

Retrief *et al.* (23) and Denys *et al.* (24) found that sufficient surface roughness and increase the wettability of the etched enamel surface are more important than depth of decalcification and resin penetration in the deeper places of the pores.

In the current study, the highest bond strength was obtained in those groups where acid-etch was applied before using the adhesive (TXE and PQ1E groups). This finding confirmed the efficacy of acid-etch technique for bonding of orthodontic brackets. But, these groups had higher ARI than the other two groups; although, these differences were statistically significant compared with the PQ1 group only.

There was no significant difference in bond strength of self-etch group (TXS) and the groups which acid-etch was applied. Other studies have found similar results. Pandis *et al.* (7) applied self-ligating and Edwise brackets in clinical situation using self-etch and total-etch adhesives, evaluated the failure rate over a year; They observed that the failure rate of self-etch adhesive did not exceed that of the total-etch adhesive. Dos Santos *et al.* (14) concluded that the success rate of brackets bonded with Transbond Plus is greater than when brackets are bonded with Transbond XT in clinical situation. By contrast, other researches demonstrated that the bond strength of self-etch adhesives is significantly lower than that of acid-etch technique (25,26). Scougall Vilchis *et al.* (28) observed that although the bond strength achieved through the application of Transbond Plus is significantly less than when Transbond XT is applied, this value is still more than the minimum bond strength needed for bonding the brackets as expressed in Reynold’s study (27). So, the major advantages of the application of self-etch adhesives for bonding the brackets could be described as: reducing procedure steps and the applicationtime, leaving fewerres in remnants after debonding of brackets,which means decreasing the time required for enamel polishing as well as the extent of enamel loss.

In this study, the bond strength presented in the PQ1 group (filled-adhesive without etch) had no statistically significant difference with that of the TXS group, although it was significantly lower than the bond strength of two other groups. Bishara *et al.* (12) stated that the clinically acceptable bond strength was achieved when acidic primer was used with highly-filled adhesive (77%), while the bond strength of the adhesive with lower filler content (10%) was not acceptable. In accordance with this observation, other studies found that increasing the filler content of adhesive can lead to increased bond strength and ARI (19,20).

The PQ1 adhesive is composed of: HEMA, methacrylic acid, phosphate monomer, ethanol, Bis-GMA, TEG DMA, 40% filler and camphorquinone. One reason for the acceptable bond strength obtained from this adhesive is the existence of an organic acid in its composition,one which may affect enamel surface when this adhesive is rubbed on the surface for 10 seconds. This adhesive also has antibacterial properties which may inhibit the formation or progression of caries. Furthermore, the radiopaque properties of PQ1 adhesive facilitate detection of the remnants of this adhesive from enamel, which can ultimately result in less damage to the enamel. The average value of ARI in the PQ1 group was significantly less than those of the other three groups. High cohesive strength of this adhesive may be a reason for lower value of ARI.

Even though sufficient bond strength is necessary for successful treatment in clinical situations, it should be kept in mind that the bonding of brackets is temporary and once the treatment is completed, they should be removed from the tooth surface. Higher bond strength can increase the extent of enamel damages and cracks, the amount of resin remnants on the enamel surface, and therefore the time needed to remove these remnants from enamel surface, and also the probability of enamel damage during removal of these particles. Arthun *et al.* (29) and Retief *et al.* (23) believed that the maximum bond strength of bonded brackets should be less than the fracture strength of the enamel, which is around 14 MPa, and in the present study, although the mean SBS value of all the groups was above this value, no enamel fractures were observed after debonding of the brackets. It should be considered that in addition to factors such as the application of acid-etch and the type of adhesive, the methods used for debonding the brackets can affect the value of ARI.

According to the results of this study, applying the PQ1 adhesive without acid-etch can provide sufficient bond strength for bracket bonding while leaving the minimum amount of resin remnants after debonding of brackets.

## Conclusions

The application of filled adhesive without acid-etch not only provides sufficient bond strength for bracket bonding, but also results in minimum resin remnants.

Due to the limitations of this study, ie, only one filled-adhesive was used and compared, these results may not be applicable to filled adhesives. The authors suggest evaluating the bond strength of different filled adhesives without applying acid-etching. They also recommend investigation of the bond strength of these adhesive after thermocycling.
